# In Vitro Studies on the Degradability, Bioactivity, and Cell Differentiation of PRP/AZ31B Mg Alloys Composite Scaffold

**DOI:** 10.1155/2017/5763173

**Published:** 2017-03-01

**Authors:** Jian Zou, Zhongmin Shi, Hongwei Xu, Xiaolin Li

**Affiliations:** Shanghai Jiaotong University Affiliated Sixth People's Hospital, Shanghai 200233, China

## Abstract

In recent years, more and more methods have been developed to improve the bioactivity of the biodegradable materials in bone tissue regeneration. In present study, we used rat mesenchymal stem cells (rMSCs) to evaluate the outcomes of Mg alloys (AZ31B, Magnesium, and Aluminum) and Platelet-rich plasma (PRP)/Mg alloys on rMSCs biocompatibility and osteogenic differentiation. Water absorption experiments indicated that both bare AZ31B and PRP/AZ31B were capable of absorbing large amounts of water. But the water absorption ratio for PRP/AZ31B was significantly higher than that for bare AZ31B. The degradability experiments implied that both samples degraded at same speed. rMSCs on the surface of AZ31B distributed more and better than those on the AZ31B scaffold. In ALP activity experiment, the activity of rMSCs on the PRP/AZ31B was markedly higher than that on the AZ31B scaffolds on the 7th day and 14th day. qRT-PCR also showed that OPN and OCN were expressed in both samples. OPN and OCN expression in PRP/AZ31B sample were higher than those in bare AZ31B samples. In summary, the in vitro study implied that AZ31B combined with PRP could remarkably improve cell seeding, attachment, proliferation, and differentiation.

## 1. Introduction

How to treat large bone defect effectively is a great challenge for the orthopedic surgeon. More and more osteoconductive scaffolds with good biodegradability and mechanical properties have been developed to repair bone defect. In recent years, more and more research methods have been dedicated to improve the bioactivity of the biodegradable materials in bone tissue regeneration [1]. Nevertheless, these biomaterials have no adequate strength or a good elastic modulus and cannot be used as a load-bearing application. Magnesium (Mg) and its alloys are one kind of fantastic degradable materials, which has excellent biocompatibility, higher strength, applicable mechanical properties, and natural bone density [2–5]. Moreover, Magnesium is one type of essential elements for human being and active bone cells in the human body [4, 6]. This means that Mg can serve as a biodegradable scaffold in the human body and can be dissolved, consumed, or absorbed gradually. It is also proved that Mg can enhance bone cell adhesion and has no negative effect on cell growth or differentiation [5]. However, the main disadvantage of pure Magnesium as biodegradable scaffold is its insufficient corrosion resistance. Hydrogen gas which is produced rapidly by Magnesium may lead to an alkaline poisoning effect and delay the tissue healing [7]. On the other hand, biomaterial scaffolds should maintain the strength to support the bone and tissue structural integrity before complete degradation in the whole healing process. However, Magnesium has inadequate strength. Though many alloys such as Aluminum and Zinc have been alloyed with Magnesium in order to improve the corrosion resistance and mechanical strength [8–10], most studies reported that the Mg alloys have negative effects on the mineralization and the bone cells' activity in vitro [11, 12].

Platelet-rich plasma (PRP) can be easily obtained from autologous blood. PRP has been used to treat wound healing, bone nonunion, and tendinitis for decades due to high concentration of platelets in plasma after special protocol. PRP, which is always considered as a bioactivator of growth factors and used as bone and tissue biomaterials, is plenty of growth factors, including platelet-derived growth factor (PDGF), transforming growth factor (TGF-b), basic fibroblast growth factor (bFGF), insulin-like growth factor (IGF), and vascular endothelial growth factor (VEGF) [13–15]. The purpose of using PRP is to create a better healing environment by concentrating the plasma components from an individual' s blood and reinjecting into the same individual at the site of injury.

Rat mesenchymal stem cells (rMSCs) are able to differentiate into various types of cells like osteoblasts, lipocytes, and so on [16]. So they are widely used as stem cells and seed cells on biomaterial scaffolds to evaluate the properties in tissue engineering [17–19]. In present study, we used rMSCs as seed cells to evaluate the effects on adhesion, viability, proliferation, and osteogenic differentiation of rMSCs on Mg alloys and PRP/Mg alloys in vitro. The alloys we used in this study are the alloy Mg-3Al-0.8Zn-0.4Mn (AZ31B).

## 2. Materials and Methods

### 2.1. Preparation of PRP

Heart blood was drawn from SD rats weighing 250–300 g. PRP was produced by double centrifugation process as described by Yuan et al. [20]. 4 mL heart blood was obtained from every rat and transferred to the sterile tube. The tubes were then centrifuged in the centrifuge (TD4, Shanghai Kait Instruments Factory, Shanghai, China) at the speed of 2500 rpm for 15 min at 4°C. Then there were three layers in the tube, including erythrocytes at bottom, the platelet-rich plasma at middle, and platelet-poor plasma at top. The erythrocytes at bottom were removed; then the top two layers of plasma were recentrifuged at the speed of 2000 rpm for 15 min. After that, the blood samples were separated into two layers: serum at top and the platelet-rich plasma at bottom. After discarding the top serum, the remaining 0.5 mL of plasma with rich platelets was PRP. Platelet counts of PRP and whole blood were analyzed in the hemacytometer (XS-2100, Mindray, China).

### 2.2. Preparation of AZ31B and PRP/AZ31B Scaffold

The preparation of bare AZ31B and PRP/AZ31B scaffold was under the sterilized condition. 0.4 mL PRP and Porous AZ31B (Ø6 × 2 mm) scaffold samples were put into one tube together. The tube was then spun at a speed of 2500 rpm for 5 min at 4°C. Due to the centrifugal force, PRP was mixed well with AZ31B scaffold. In addition, the interior porous structure of AZ31B scaffold were full of PRP. At final, the AZ31B scaffold full of PRP was immersed into a solution containing 0.8 IU thrombin and 1 mL CaCl2 for less than 3 seconds. After picking up from mentioned solution, the PRP/AZ31B scaffold was obtained.

### 2.3. Scaffold Characterization

The morphology of the bare AZ31B and PRP/AZ31B scaffold was observed with an scanning electron microscope (SEM, S-3400N, HITACHI, Japan).

### 2.4. Water Absorption of AZ31B and PRP/AZ31B

The original weight of bare AZ31B and PRP/AZ31B samples with sizes of Ø 6 × 2 mm was measured with an electronic scale (CP64C, OHAUS, America). Then, the samples were immersed in water for 0.5, 6, 12, 18, 24, 30, 36, 42, and 48 h. At the selected time points, the samples were removed from solution and the weight again was measured. The ratio of water absorption was defined as weight increase/original weight ×100%.

### 2.5. Degradation of AZ31 and PRP/AZ31 in the Tris-HCl Solution

The measurement of original weight of samples has been mentioned. Ø 6 × 2 mm AZ31B and PRP/AZ31B samples were soaked into the sealed bottles with Tris-HCl solution to evaluate the degradation behavior. The bottles were placed at a temperature of 37°C and a humidity of 50%. The Tris-HCl solution was refreshed every three days. After 4, 14, 28, 42, 56, and 70 days of immersion, the samples were removed from the solution and cautiously washed by distilled water. After that, the samples were dried in an oven at a temperature of 100°C for 8 h and the weight was measured again. The ratio of degradation was defined as weight decrease/original weight ×100%.

### 2.6. rMSCs Culture, Seeding, and Attachment

The AZ31B and PRP/AZ31B scaffold samples were cut into Ø 6 × 3 mm small columns. The small columns were sterilized by gamma radiation (15 kGy) and prewetted with DMEM solution. Then, they were put into 48-well plates.

rMSCs were purchased from Sciencell Research Laboratories (Carlsbad, CA, USA) and were cultured in differentiation in accordance with manufacturer's protocols. Cells after the four passage were seeded at density of 50,000 cells/mL and were cultured in MEM*α*. rMSCs were cultured on AZ31B and PRP/AZ31B scaffolds for 7 days before bone differentiation.

### 2.7. Cell Morphology, Distribution, and Proliferation

To observe the inside distribution and penetration of rMSCs, the scaffold samples were cut along the middle line. For confocal laser scanning microscope (CLSM) observation, the samples prepared as described above were rinsed slightly with PBS and were fixed in 4% paraformaldehyde for 10 min. Following pretreatment using Triton X-100 (0.5% v/v) for 5 min, the cells were stained with rhodamine-phalloidin (Sigma) and Hoechst 33258 (Sigma) in the dark for 30 min and 5 min, respectively. The Factin and cell nuclei were observed by CLSM (LSM 510 meta; Zeiss, Germany). MTT assay (Sigma, USA) was used to assess cell proliferation in accordance with the manufacturer's protocols.

### 2.8. ALP Activity

After 1, 3, 7, and 14 days of culture in osteogenic medium, Para-nitrophenyl phosphate (p-NPP) (Sigma) was used to measure alkaline phosphatase (ALP) activity of rMSCs cells in accordance with the manufacturer's protocols. Measuring the Synergy2 (Biotech) microplate reader of the para-nitrophenol (p-NP) was used to calculate the ALP activity.

### 2.9. Quantitative Real-Time Polymerase Chain Reaction (qRT-PCR)

After culturing in differentiation media, RNA extraction was performed using the TRIZOL reagent (Invitrogen) in accordance with the manufacturer's protocols. The concentration and purity of RNA were calculated by measuring the absorbance at 260 and 280 nm. The reverse transcription kit (Sciencell Research Laboratories, Carlsbad, CA, USA) was used to perform reverse transcription in accordance with the manufacturer's instructions.  Table 1 shows the Primers of mouse GAPDH, osteopontin, and osteocalcin in the qRT-PCR experiments.

### 2.10. Statistical Analysis

All quantitative data were displayed as the mean ± standard deviation (M ± SD) and analyzed with SPSS statistical software program (version 12.0.0, SPSS, IBM). Wilcoxon rank sum test was carried out to determine the significance. Significant differences were defined as *P* value less than 0.05.

## 3. Results

### 3.1. Material Characterization

**Figure 1:**
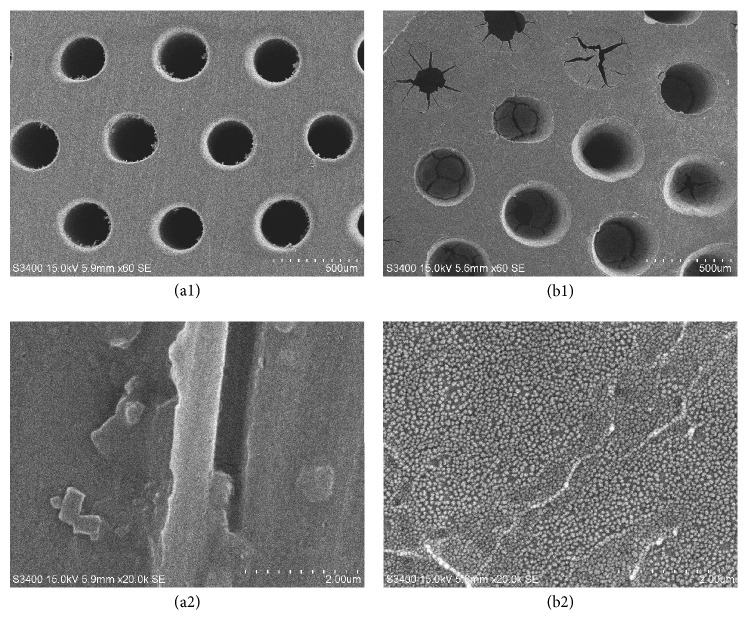
The surface morphologies of the bare AZ31B (a1, a2) and PRP/AZ31B (b1, b2) scaffolds.

The platelet concentrations of whole blood and PRP were (0.38 ± 0.04) ×10^9^/mL and (1.21 ± 0.06) ×10^9^/mL, respectively. It showed that the platelet concentration of PRP was almost 3.2 times that of the whole blood. The morphologies of bare AZ31B and PRP/AZ31B scaffold were given in Figure 1. The bare AZ31B was highly porous with regular distributed macropores with an average diameter of 300 um (Figures 1(a1) and 1(a2)). Abundant PRP sheets were filled to the macropores of the AZ31B scaffold after the centrifugation. Large quantities of platelets were observed on the surface of AZ31B (Figures 1(b1) and 1(b2)).

### 3.2. Water Absorption and Degradability

**Figure 2:**
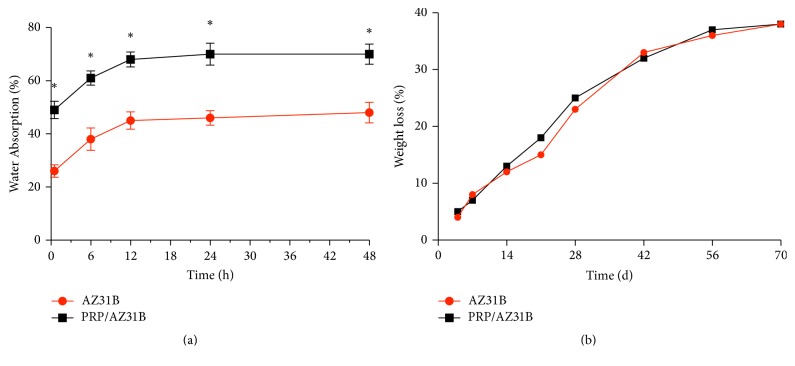
Water absorption (a) and weight loss (b) of AZ31B and PRP/AZ31B soaked in Tris-HCl solution (*n* = 6). ^*∗*^*P* < 0.05.

Figure 2(a) showed the water absorption ratio of AZ31B and PRP/AZ31B samples at different time points. Both AZ31B and PRP/AZ31B continued to absorb water as time goes by. The water absorption ratios of bare AZ31B after soaking in water for 0.5 h and 12 h were 26% and 45%, respectively. The ratios of PRP/AZ31B were 49% and 68%. These results proved that both AZ31B and PRP/AZ31B were capable of absorbing large amounts of water. But the water absorption ratio for PRP/AZ31B was significantly higher than that for bare AZ31B.

The results of degradation behavior of AZ31B and PRP/AZ31B were different to those of water absorption. After immersion in Tris-HCl solution, both samples showed weight loss over time. The weight loss curve in Figure 2(b) showed that the weight loss ratio of the AZ31B was no different to that of PRP/AZ31B at any selected time point. The weight loss ratio in both samples was less than 40 wt% at 70 days. These results implied that both PRP/AZ31B and AZ31B degraded at same speed.

### 3.3. Cell Morphology and Distribution

**Figure 3:**
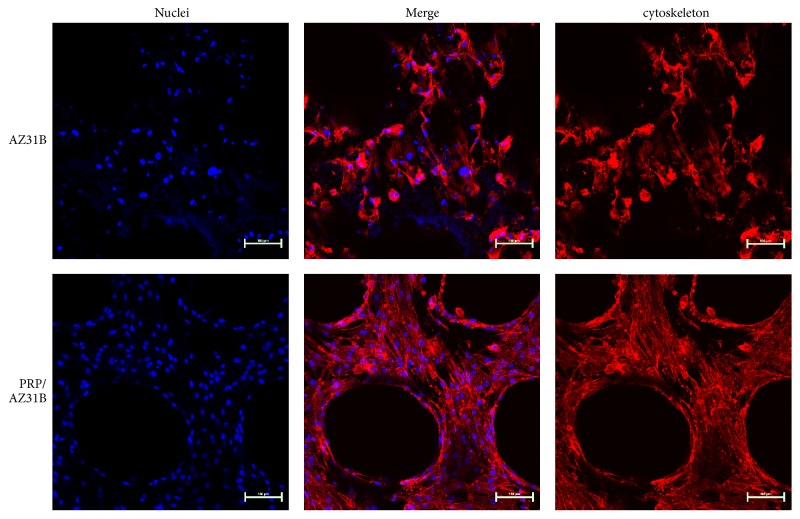
The CLSM images of cell morphology on both scaffolds after 7 days of incubation.

Figure 3 demonstrated the CLSM photographs of rMSCs which had been cultured for 8 days on the bare AZ31B and PRP/AZ31B scaffold. The cells distributed regularly on the cut surface of AZ31B and PRP/AZ31B scaffold. More rMSCs were observed on the surface of PRP/AZ31B scaffold in comparison to those on AZ31B. Moreover, the rMSCs on the PRP/AZ31B scaffold distribute better than those on the bare AZ31B scaffold. There was no cell spread in the macropores in both samples. These results implied that the rMSCs were able to migrate to the inside of the scaffold smoothly as the culture time goes by. Plenty of PRP did not compromise the cell infiltration but improved the cell proliferation.

### 3.4. Cell Proliferation

**Figure 4:**
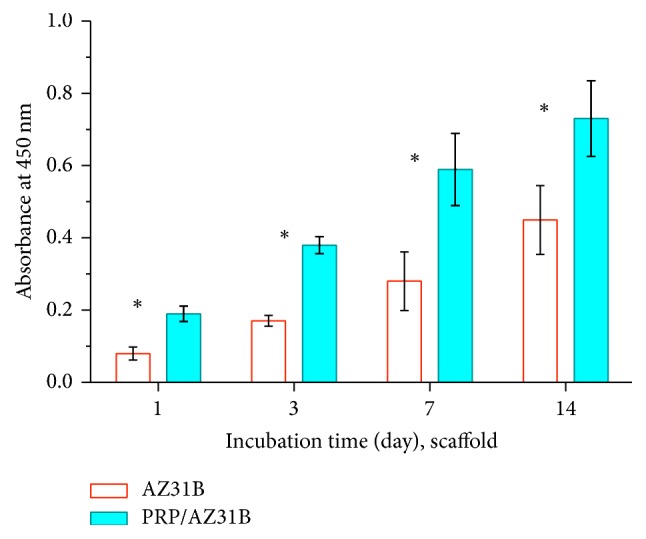
The rMSCs proliferation on the AZ31B and PRP/AZ31B scaffolds (*n* = 6). ^*∗*^*P* < 0.05.

The cell proliferation on both AZ31B and PRP/AZ31B scaffolds at 1, 3, 7, and 14 d was manifested in Figure 4. On the first day of culture, the rMSCs on the PRP/AZ31B scaffold were apparently more than those on the AZ31B scaffold. As the time goes by, the cells on both scaffolds proliferated well. But comparing with the cells on the AZ31B, those on PRP/AZ31B were significantly more (*P* < 0.05).

### 3.5. ALP Activity

**Figure 5:**
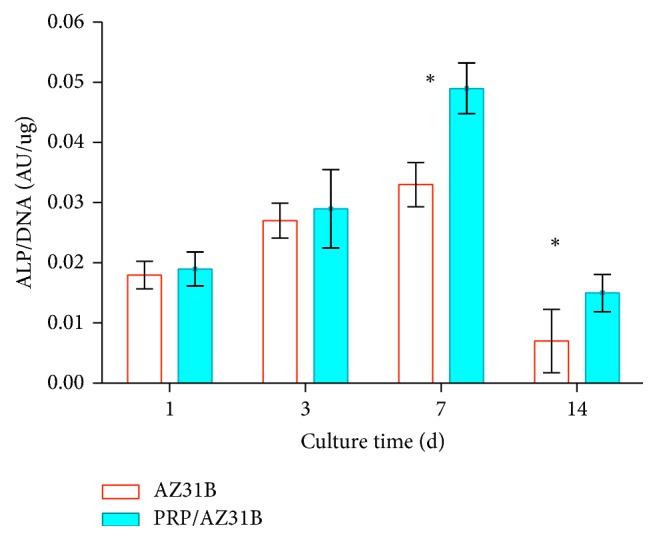
The ALP activity of rMSCs cultured on the AZ31B and PRP/AZ31B scaffolds (*n* = 6). ^*∗*^*P* < 0.05.

Figure 5 displayed the ALP activity of rMSCs which were cultured on both AZ31B and PRP/AZ31B scaffolds. On the 1st and 3rd day, the ALP activity was not different in AZ31B and PRP/AZ31B scaffolds. On the 7th day and 14th day, the ALP activity of the PRP/AZ31B was markedly higher than that of the AZ31B scaffolds (*n* = 6, *P* < 0.05). The curve also demonstrated that ALP activity in both samples increased from the 1st day to 7th day and then decreased to the 14th day.

### 3.6. qRT-PCR

**Figure 6:**
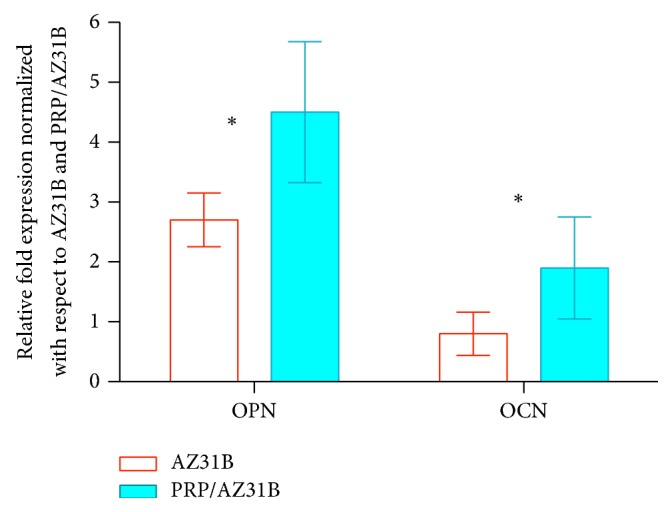
qRT-PCR gene expression data show the expression of osteoprotein (OPN) and osteocalcin (OCN) of rMSCs cultured on coated samples normalized with respect to cells cultured on bare AZ31B and AZ31B/PRP. ^*∗*^*P* < 0.05.

qRT-PCR was performed after the rMSCs were cultured in differentiation media for 14 days.  Figure 6 showed that both OPN and OCN levels in PRP/AZ31B samples were significantly higher than those in bare AZ31B sample (*P* < 0.05).

## 4. Discussion

In the past few decades, traditional materials were widely used in orthopedic surgeries. However, Mg and Mg alloys nowadays were considered to be newly developed components of biomaterial scaffold for tissue engineering and prevail gradually. Recent reports have suggested that Mg and Mg alloys are accepted and used as biodegradable osteoconductive scaffold owing to their desirable biocompatibility and higher strength. In our current study, a PRP/Mg alloys composite scaffold with unidirectional pores was developed to evaluate the in vitro efficacy on rMSCs adhesion, viability, proliferation, and osteogenic differentiation.

PRP is a plasma enriched with platelet and contains a variety of growth factors including TGF-*β*, PDGF, VEGF, BMPs, and cytokines [21] and therefore plays a crucial role in osteogenesis, angiogenesis, and soft tissue healing stimulation. Furthermore, various concentrations of growth factors can be detected once the platelet concentration of PRP is controlled [22]. Once the cells are cultured in the environment with PRP, the fibrin and a variety of growth factors released from platelets have synergistic effect on cells' behavior. Namazi and Kayedi [23] indicated PRP may have a significant therapeutic effect on pain relief at rest and alleviate function difficulties including specific and usual activities in patients with scaphoid fractures in a study with 14 patients enrolled. Ghaffarpasand et al. [24] determined that application of PRP along with autologous bone graft in the site of nonunion of long bone after intramedullary nailing or ORIF resulted in high cure rate, short duration of healing, low rate of limb shortening, and mild postoperative pain. Malhotra et al. [25] injected autologous platelet-rich plasma to 94 long bone nonunion patients and found that 82 patients got united at the end of 4 months. They demonstrated that PRP was a safe and effective method for nonunion therapy. In this study, the PRP/AZ31B scaffold not only prevented the cells from peeling off the scaffold, but also provided more bioactive surface areas to promote cell adhesion. Therefore, the cell seeding of the scaffold was markedly improved. The cell attachment, proliferation, and differentiation on the PRP/Z31B scaffold notably ameliorated as well when compared with those of bare AZ31B scaffold due to synergistic effect of both fibrin and other growth factors released from the platelets.

In present study, the water absorption experiment showed that large amount of water was absorbed in both AZ31B and PRP/AZ31B with the water absorption ratio of the PRP/AZ31B (57%) almost twice higher than that of bare AZ31B scaffold (33%). The infiltration of PRP into AZ31B significantly increased in water absorption test, suggesting that PRP had much specific hydrophilic surface area and enhanced alloy's capacity to absorb water. These results inferred that the PRP/AZ31B, which had specific surface and remarkable rapid and efficient capability of water absorption, could be used as a biomaterial scaffold to load target drugs, cells, and other biomolecules with therapeutic effect [26]. As candidate biomaterials for bone repair and remodeling, these implants and scaffolds must feature with appropriate degradable property and can be gradually replaced by new bone tissue in in vivo models [27]. The weight loss of both AZ31B and PRP/AZ31B in Tris-HCl solution increased with incubation time and reached up to 38% after 70 days of incubation. Little variance of degradability was found between two samples. These results suggested that PRP/AZ31B could not only improve the cell adhesion and proliferation, but also have no adverse effect on degradability. Bone tissue regeneration is attributable to PRP/AZ31B's favorable biochemical properties to a large extent. [28].

In our current study, the proliferation of rMSCs on the surfaces of both PRP/AZ31B and AZ31B was evaluated by MTT assay. The results showed that PRP/AZ31B was in favor of the rMSCs proliferation at every time point, indicating that PRP/AZ31B with good cytocompatibility had no toxicity for cell growth and proliferation. In addition, ALP activity, which is one of the most widely used markers for detection of osteoblastic differentiation, is also a generally used to measure the osteoblast differentiation in the early stage [29]. The results in our study revealed that the ALP activity of the cells on both samples was time-dependent and ALP activity in PRP/AZ31B was higher than that in bare AZ31B sample at the time points of 7 and 14 d. These results also indicated that the PRP/AZ31B scaffolds could improve rMSCs differentiation to some extent. The reasonable explanation was that PRP's infiltration into AZ31B could enhance cell proliferation and differentiation after 3 days' culture while high concentration of PRP and multipore structure of AZ31B provide more growth factors and therefore facilitated cell growth as seen in the experiments.

The cytocompatibility is a very critical criterion to assess cell spread on the surface of the substrate [30]. In our study, the results suggested that the rMSCs cells extended and spread well on the bare AZ31B and PRP/AZ31B surface and formed tight attachment to substrate after 24 h of culture, indicating that both AZ31B and PRP/AZ31B had no adverse effect on cell morphology and viability. Obviously, the process of cells' spread on PRP/AZ31B surface was much faster than that on bare AZ31B surface due to complex effect of a variety of growth factors of PRP. Therefore, our results suggested that PRP/AZ31B with plenty of bioactive factors was favorable for cell attachment, growth, and spread with good cytocompatibility. PRP/AZ31B may play a role in promoting new bone formation and accelerating surrounding tissue healing process when implanted in vivo.

There is no new concerned toxicity when bare AZ31B and PRP/AZ31B are applied to support rMSCs proliferation. Osteocalcin (OCN) and osteopontin (OPN), which were encoded by osteogenic genes, were studied on both samples to investigate the capability of AZ31B and PRP/AZ31B to promote the differentiation of rMSCs. OPN, encoded by SPP1 gene, is a noncollagenous, extracellular structural protein which is well-known to accelerate wound healing and bone mineralization [31]. Because OPN has played an important role in mineralization and is able to adjust cell attachment and adhesion on the biomaterials, it has been used as biomarker for osteogenic differentiation in the early stage [32, 33]. OCN, which is secreted by osteoblasts and plentiful in bone, is the other biomarker for osteogenic differentiation and metabolic regulation. In contrast, OCN is a late-stage biomarker [34]. It has been proved that high serum OCN levels are correlated with high bone mineral density (BMD) in adults. Many researches stated that osteocalcin is used as a preliminary biomarker to evaluate the efficacy of antiosteoporosis drug. In our current study, qRT-PCR was performed after 14 days of cell culture. Though high levels of OPN and OCN were expressed in both samples as anticipated, OPN and OCN expressions in PRP/AZ31B sample were higher than those in bare AZ31B samples. The results revealed that PRP/AZ31B may apply better biochemical microenvironment for osteogenic differentiation, resulting in rapid progress of soft tissue healing and bone remodeling.

## 5. Conclusion

In this study, we developed a porous PRP/My alloys composite scaffold to evaluate the effects on rMSCs adhesion, viability, proliferation, and osteogenic differentiation. Water absorption experiments indicated that both bare AZ31B and PRP/AZ31B were capable of absorbing large amounts of water. But the water absorption ratio for PRP/AZ31B was significantly higher than that for bare AZ31B. The degradability experiments implied that both samples degraded at same speed. rMSCs on the surface of AZ31B distributed more and better than those on the AZ31B scaffold. In ALP activity experiment, the ALP activity of the PRP/AZ31B was markedly higher than that of the AZ31B scaffolds on the 7th day and 14th day. qRT-PCR also showed that OPN and OCN were expressed in both samples. OPN and OCN expressions in PRP/AZ31B sample were higher than those in bare AZ31B samples. In summary, the in vitro study implied that AZ31B combined with PRP could remarkably improve cell seeding, attachment, proliferation, and differentiation.

## Figures and Tables

**Table 1:** qRT-PCR primer sequences used for mouse MSC.

	Forward primer (5′ - 3′)	Reverse primer (5′ - 3′)
GAPDH	GATTTGGCCGTATCGGAC	GAAGACGCCAGTAGACTC
Osteocalcin	CATGCCAGGTCACCAAAT	GCCCCAAGGCCGCTTCTT
Osteopontin	ACTCAGATGCTGTAGCCA	TTTCATTGGAGTTGCTTG
